# Tumor microenvironment-aware, single-transcriptome prediction of microsatellite instability in colorectal cancer using meta-analysis

**DOI:** 10.1038/s41598-022-10182-3

**Published:** 2022-04-15

**Authors:** Mi-Kyoung Seo, Hyundeok Kang, Sangwoo Kim

**Affiliations:** Department of Biomedical Systems Informatics, Brain Korea 21 Project, Yonsei University College of Medicine, Seoul, 03722 South Korea

**Keywords:** Computational models, Cancer microenvironment, Colorectal cancer

## Abstract

Detecting microsatellite instability (MSI) in colorectal cancers (CRCs) is essential because it is the determinant of treatment strategies, including immunotherapy and chemotherapy. Yet, no attempt has been made to exploit transcriptomic profile and tumor microenvironment (TME) of it to unveil MSI status in CRC. Hence, we developed a novel TME-aware, single-transcriptome predictor of MSI for CRC, called MAP (Microsatellite instability Absolute single sample Predictor). MAP was developed utilizing recursive feature elimination-random forest with 466 CRC samples from The Cancer Genome Atlas, and its performance was validated in independent cohorts, including 1118 samples. MAP showed robustness and predictive power in predicting MSI status in CRC. Additional advantages for MAP were demonstrated through comparative analysis with existing MSI classifier and other cancer types. Our novel approach will provide access to untouched vast amounts of publicly available transcriptomic data and widen the door for MSI CRC research and be useful for gaining insights to help with translational medicine.

## Introduction

Microsatellite instability (MSI) is characterized by genetic hypermutability due to impaired DNA mismatch repair (MMR) system^1^. MSI is observed in many solid tumors, including gastric, and endometrium cancers, as well as in colorectal cancer (CRC, approximately 15%)^2,3^. Exhibiting prognostic and predictive features of a high tumor mutational burden, a high neoantigen load, and an immune-active tumor microenvironment (TME) characterized by high levels of tumor-infiltrating lymphocytes and overexpression of immune checkpoint molecules, cancers with MSI are known to be great candidates for immune checkpoint inhibitors (ICIs) treatment, such as pembrolizumab and atezolizumab (anti-PD-1 and anti-PD-L1 monoclonal antibody, respectively)^4,5^. MSI status is meaningful as a predictive indicator for cancer treatment and as a prognostic determinant, identifying a patient's MSI status is essential in clinical setting and research areas.

With recent escalation of its importance in CRC, it has been explored from publicly obtained samples, such as The Cancer Genome Atlas (TCGA) and Gene Expression Omnibus (GEO) database, resulting in numerous studies which broaden our understanding in MSI and expand therapeutic options for MSI CRC patients^6^. However, prior to utilization, MSI status information must be provided beforehand by quantifying the extent of genomic events in microsatellite loci, at genomic level, using the Bethesda Panel, a PCR-based five marker panel, or by examining the loss of mismatch repair proteins using immunohistochemistry (IHC) at the protein level^1^. Additionally, with recent advances in next-generation sequencing (NGS) technology, MSI predictors, such as MANTIS^7,8^, MSIsensor^9^, and MOSAIC^10^, MSICare^11^ have been developed to extract MSI status from whole exome and whole genome sequencing data. However, assigning the MSI status from expression data had not been possible until a k-Nearest Neighbors (k-NN, k = 5) classifier called preMSIm using 15 gene-set for pan-cancer was recently proposed^12^ and several other attempts which had been made, although the software has not been made readily available^13,14^.

The preMSIm has constructed as a pan-cancer MSI predictor by utilizing three MSI dominant carcinomas as training data^12^, but it did not reflect the distinct expression profiles of its cancer of origin. Furthermore, individual MSI tumors have unique tumor microenvironment (TME) and molecular pathway characteristics. For example, immune inflamed MSI microenvironment could be characterized by higher infiltration of anti-tumorigenic immune cells, such as adaptive immune cells (T and B lymphocytes) and innate immune cells (dendritic cells, macrophages, and natural killer cells) than immune desert MSS tumors, and, in CRC, when mutations or activation of MYC and RAS pathways occur, chemokine *CCL9* is expressed and an immunosuppressive environment is established, which prevents enrichment of cytotoxic NK cells and T cells around the tumor^15^. There are also comprehensive immune and stromal cell type studies using the transcriptome-based cell-type quantification method and pathway studies according to MSI status in colorectal cancer^16^. IFN-γ and CD8 T effector gene signatures were highly activated in the MSI group compared to the MSS group, suggesting that this phenomenon is also associated with CD8 cell infiltration and upregulation of PD-L1 and PD-L2 and p-STAT1^17^. This suggests that MSI and MSS constitute a unique TME that will contribute to immunotherapy, thus implying the importance of understanding TME as well as tumor characteristics. Therefore, transcriptome based MSI predictor which integrates both TME and molecular pathway characteristics in CRC is needed.

Here, in this study, we have developed an enhanced single-sample MSI classifier called MAP (Microsatellite instability Absolute single sample Predictor) that integrates transcriptomic characteristics of TME and molecular pathways to predict MSI in CRC. Our TME and molecular pathways aware predictor will open a way to utilize CRC expression data to elucidate MSI CRC. Hence, massive amounts of publicly available expression data without MSI status will be utilized to drive valuable MSI CRC research through our novel approach.

## Results

### Overview of MAP development

**Figure 1:**
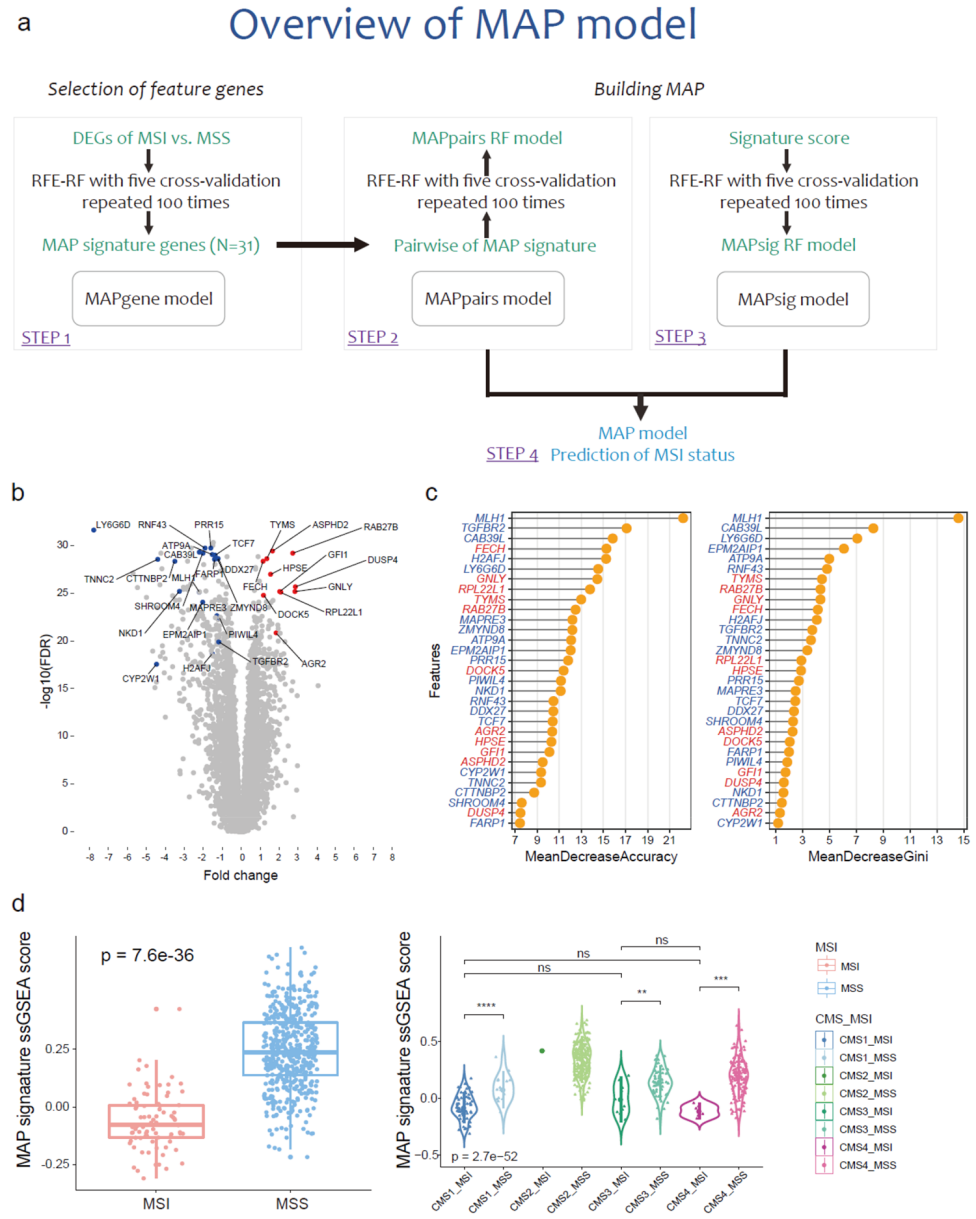
Overview of the MAP model and designing the MAP signature from RFE-RF analysis of gene expression data. (**a**) Overview of the MAP model. MAP was developed through a workflow consisting of four strategies. (1) identification of the MAP signature (MAPgene model); (2) modeling based on pairwise gene expression of the MAP signature genes (MAPpairs model); (3) modeling based on ssGSEA scores of cancer-, molecular-, TME-, and immune-related signatures (MAPsig model); and (4) post-refinement of the final model and prediction of MSI status. (**b**) A volcano plot for DEGs between MSI and MSS samples. The x axis represents log_2_ fold changes in gene expression data for MSI versus MSS samples. Colored dots are significant DEGs in MAP signature; red and blue indicate up- and downregulated genes, respectively. (**c**) The importance of 31 features is based on accuracy and Gini index scores. The mean decrease in accuracy is a measure of how much influence it has in improving classification accuracy. The mean decrease in Gini is a measure of how impurity can be reduced by features used when separating nodes. The genes with red and blue colors indicate up- and downregulated genes in MSI, compared with MSS, respectively. (**d**) MAP signature. A box-plot of MAP signature ssGSEA scores according to MSI status (left) and CMS-MSI and MSS subtypes (right). The dots represent samples. MAP signature scores differ significantly between MSI and MSS samples independent of CMS subtypes. CMS2-MSI did not confirm statistical significance because the number of samples was small. * *P* < 0.05, ** *P* < 0.01, *** *P* < 0.005. DEG; differentially expressed gene, MSI; microsatellite instability, MSS; microsatellite stability, RFE-RF; recursive feature elimination-random forest, CMS; consensus molecular subtype, ssGSEA; single-sample gene set enrichment analysis, FDR; false discovery rate.

We developed MAP, a method that can predict MSI status leveraging transcriptome data of colorectal cancer samples. As an MSI single sample predictor (SSP), the MAP model was developed with the following four components (Fig. 1a): (1) identification of the MAP signature (MAPgene model); (2) modeling based on pairwise gene expression of the MAP signature genes (MAPpairs model); (3) modeling based on ssGSEA scores of cancer-, molecular-, TME-, and immune-related signatures (MAPsig model); and (4) post-refinement of the final model and prediction of MSI status. To develop an SSP of MSI status without relying on a relative approach (e.g., comparing a patient’s data with other samples) and with limited platform bias, we constructed a recursive feature elimination-random forest (RFE-RF) model (MAPpairs model) with pairwise gene comparisons, leveraging an informative gene-set (MAP signature from the MAPgene model), rather than gene expression profiles, on a training dataset. In brief, RFE trains the model, ranks the features, and selects features through the process of repeatedly removing lower-ranked features^18^. The method of building a model by selecting features with the RFE method based on the RF algorithm is called RFE-RF^18^. We built another RFE-RF model (MAPsig model) based on ssGSEA scores for 101 signatures to reflect the degree of activity of cancer-, immune- and TME-related signatures of the samples. To select the best RFE-RF model from the parts mentioned above, we evaluated the area under the receiver operating characteristic curve (AUC) and confirmed the model performance in validation datasets (Table S1). Finally, at the post-refinement stage, an integrated MAPpairs and MAPsig model was used to precisely predict MSI status. We named this final model MAP and evaluated its accuracy, kappa, sensitivity, specificity, F1, and balanced accuracy in the validation datasets (Table S2).

### MAP signature

To minimize the size of the informative gene-set utilized in the MAPgene model, we, first, identified differentially expressed genes (DEGs) between MSI and MSS samples using the Wilcoxon rank-sum test. We assessed 718 DEGs with criteria of *P* < 0.001 and |log_2_ fold change|> 1, and selected a gene-set of 31 genes by performing RFE-RF modeling with an AUC of 99.2%. We called this gene-set the MAP signature (11 up- and 20 down-regulated DEGs, Fig. 1b and Table S3). Among genes comprising the MAP signature, the *MLH1* gene, which is commonly downregulated and/or hypermethylated in sporadic MSI samples, ranked as the top feature gene, based on both accuracy (the importance of the features that improves classification accuracy) and Gini index values (the importance of the features that reduces the impurity of classification) (Fig. 1c and Table S3). We also noted that *LY6G6D*^19^ and *EPM2AIP1* genes^20^, which share a promoter with *MLH1*, were included in the gene-set. Additionally, we found that a known predictive marker for chemotherapy, thymidylate synthase (*TYMS*)^21^, was included in the gene-set, and its expression was higher in the MSI samples than the MSS samples (Fig. 1b,c). Other genes belonging to the following pathways were also included in the MAP signature: the WNT signaling pathway (*RNF43, TCF7,* and *NKD1*), Hippo signaling (*TCF7, NKD1,* and *TGFBR2*), and MAPK signaling (*DUSP4* and *TGFBR2*). Three well-known frameshift mutated genes (*DDX27, TGFBR2*, and *RNF43*) in microsatellite loci in MSI CRC were also included. In terms of MMR, 718 DEGs were initially used when constructing the MAPgene model, although three MMR genes (*MSH2, MSH6*, and *PMS2*) were not included because their statistical significance or fold change did not meet the inclusion criteria (Fig. S1).

To assess the representativeness of the MAP signature (31-genes) in reflection of MSI status, the expression patterns of the genes in the gene-set were investigated in a validation dataset, and expression patterns similar to those observed in the discovery dataset were noted. To further investigate whether the MAP signature could serve as a surrogate marker of MSI status and to evaluate its generalizability, we obtained and compared ssGSEA scores for the MAP signature in MSI and MSS tumors, as well as in MSI tumors of each of the four consensus molecular subtypes (CMSs). The general comparison between MSI and MSS samples revealed significant differences in MAP signature cores (*P* = 7.6 × 10^−36^), but not among the CMSs (Fig. 1d). This suggests that the composite genes of the MAP signature can capture MSI’s behavior-related features and discriminate between MSS and MSI status independent of CMS context.

### MAP model

**Figure 2:**
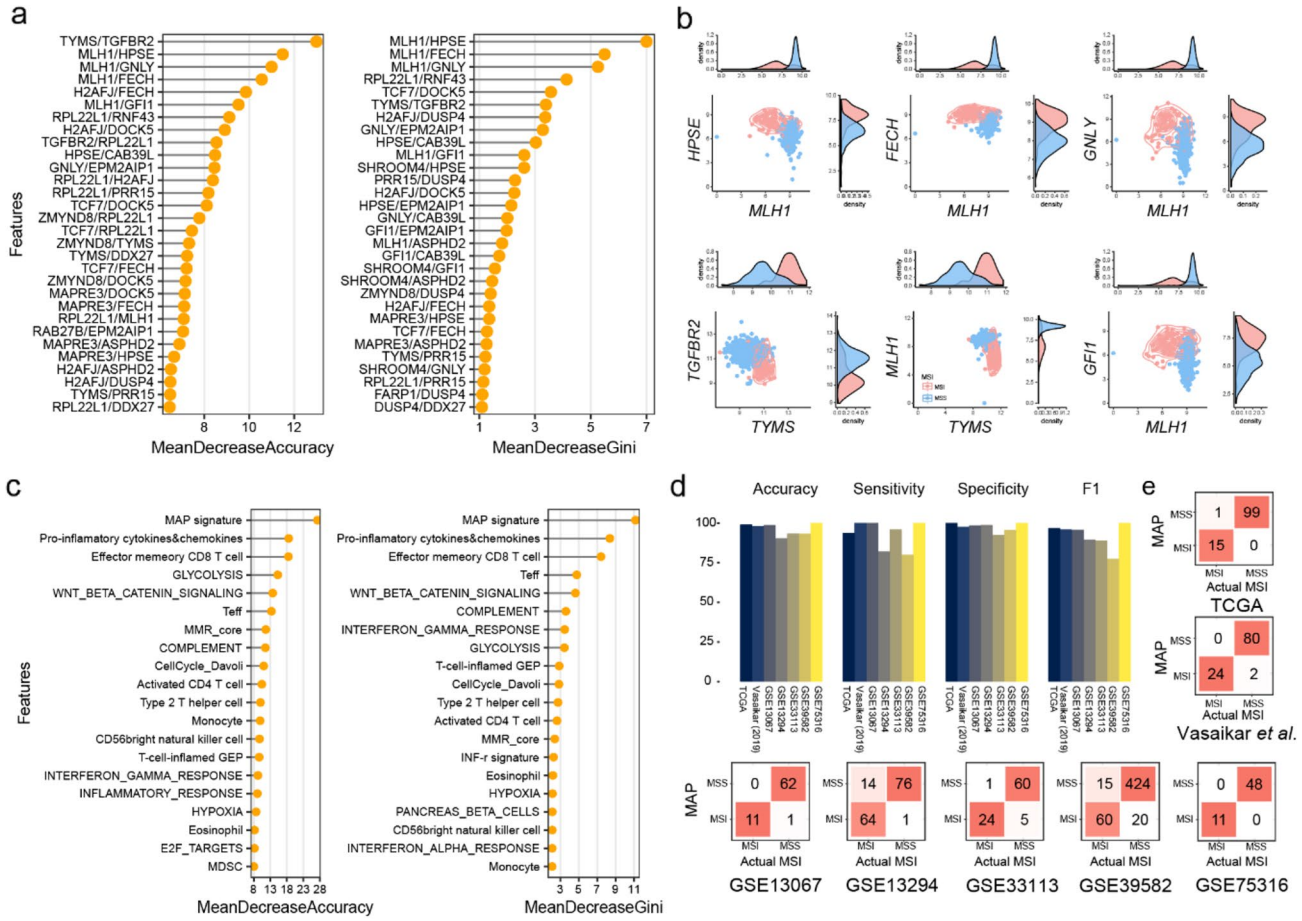
MAP model. (**a**) Top 30 important features of the MAPpairs model. The mean decrease in accuracy (left) is a measure of how much influence a feature has in improving classification accuracy. The mean decrease in Gini (right) is a measure of how impurity can be reduced by features used when separating nodes. (**b**) A scatter plot and histogram of the gene pairs. The relationship between the expression of two genes in the MSI and MSS groups can be confirmed through a scatter plot, and the expression value of each gene can be confirmed through the density plots at the upper and lower right corners. *MLH1*-related rules and *TFGBR2/TYMS* rule are shown. (**c**) Top 20 important features (signatures) of the MAPsig model. (**d**) Performance (accuracy, sensitive, specificity, and F1) of the MAP model. (**e**) Confusion matrices of the validation dataset. The actual MSI means MSI status provided in the dataset study. The red color-scale reflects percentages of class predictions against the actual class.

Although the MAPgene model built based on gene expression showed high performance, to develop a true SSP with unnormalized data that does not rely on a relative comparison among multiple samples, we employed a pairwise gene comparison approach for model building: for example, if the expression of gene A was greater than that of gene B, the sample would be assigned MSI status. An RF model with 1000 trees of such rules was constructed utilizing the RFE-RF algorithm with five-fold cross-validation repeated 100 times. Finally, the MAPpairs model, comprised of 187 rules from 465 (_31_C_2_) rules at a starting point, was selected (AUC of 99.7%). To assess its performance and reproducibility, we applied the model to internal and external RNA-seq validation datasets and obtained accuracies of 99.1% and 95.4%, respectively, indicating it to be robust and highly accurate. In the MAPpairs model, MLH1-related features (*MLH1/HPSE, MLH1/FECH*, and *MLH1/GNLY*) were the highest ranked features (Fig. 2a), and the ratios of genes (features) tend to enrich each group, and the expression value of each gene differs between groups, indicating that the ratio rules used in the MAPpairs model can distinguish the two groups well. (Fig. 2b). To investigate the features of MAPpairs further, we calculated the number of MSI and MSS samples that satisfied each rule in MAPpairs (Fig. S2). Most rules were able to classify MSI and MSS samples, and as such, they were considered to be reflective of the overall characteristics of MSI, although not all samples may show similar profiles. For example, MSI samples are known to have a loss of *MLH1* and an immunity-activated characteristic^2^, as well as high expression of thymidylate synthase (*TYMS*) (chemotherapy response-associated gene)^21^. Features of the MAPpairs model, *MLH1/GNLY* and *TYMS/MLH1*, respectively, described these MSI characteristics well (Fig. 2b), but not in all tumors. This may suggest that the MAPpairs model, a random forest classifier, captures and reflects the more complexity of MSI CRC, not just a simple single rule.

In order to complement the MAPpairs model with the characteristics of immune and TME profiles, as well as the transcriptomic profile and tumor’s characteristics of MSI, we built the MAPsig model based on molecular, cancer, immune, TME, and MAP signature scores inferred by single-sample gene set enrichment analysis (ssGSEA). The top signatures used in the final MAPsig model (44 signatures) included the MAP signature, antitumorigenic immune lymphocytes (effector memory CD8 T cell, Teff (CD8 T effector), Th2 cells, activated CD4 T cell), complement, INF-γ signatures, Wnt-β/catenin signaling, glycolysis, and cell cycle signaling (Fig. 2c). To find out the degree of activation of 44 signatures, we investigated the heatmap based on the inferred ssGSEA score. Compared to MSS, antitumorigenic immune lymphocytes, complement, glycolysis, cell cycle, and INF-γ related signatures were up-regulated in MSI, whereas MAP signature, Notch, angiogenesis, epithelial signature, and Wnt-β/catenin signaling were down-regulated (Fig. S3). To investigate the effect of MSI status on TME and oncogenic signatures, we analyzed the correlation between signature features and MSI probability of MAPsig model (Spearman correlation *P* < 0.01 and |rho|> 0.3). MAP signature, Wnt-β/catenin signaling, and epithelial signature showed the most negative correlation, suggesting that the higher these signature score values, the more down-regulation in the MSI group (Figs. S3 and S4). On the other hand, immune-related signatures such as pro-inflammatory cytokines and chemokines, type 2 T helper cells, effector memory CD8 T cells, interferon gamma, and inflammatory signatures were observed to show a positive correlation with MSI status or to show up-regulation in the MSI group (Figs. S3 and S4). The final MAP model was established after integrating the MAPpairs and the MAPsig models, and post-refinement processing was done by utilizing probability. Next, we applied the final MAP model to validation datasets to evaluate any potential overfitting and its applicability across multiple platforms. A total of 1118 samples (240 MSI and 878 MSS tumors) were tested, and MAP exhibited an average accuracy of 96.1% (95% confidence interval (CI) 94.3–98.9), a sensitivity of 93.1%, a specificity of 97.5%, and an F1 score of 92.0% (Fig. 2d,e), indicating outstanding performance and feasibility as an MSI predictor.

### MSI signatures in other cancer types

**Figure 3:**
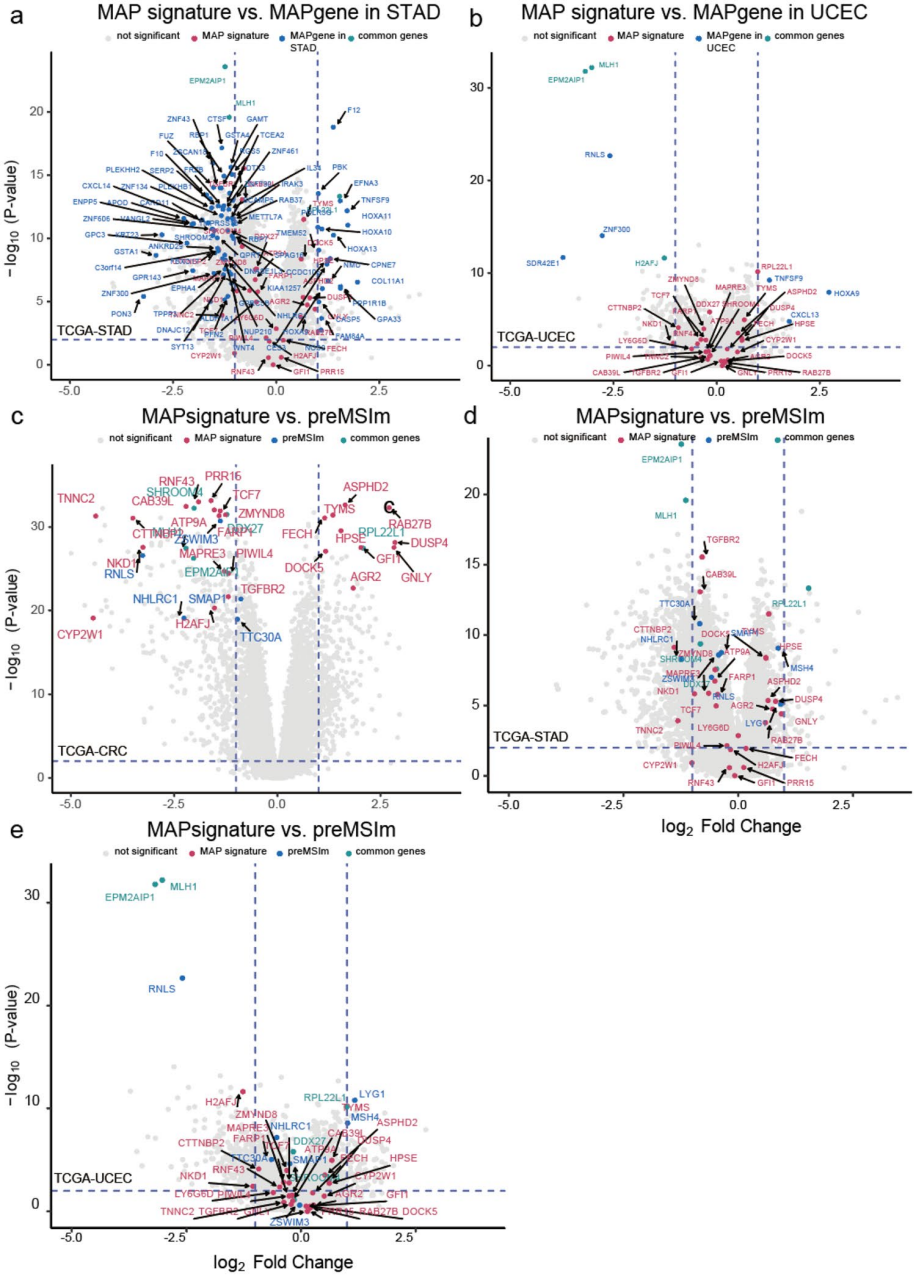
MSI signatures. (**a**) MAP signature and UCEC MSI signature on TCGA-UCEC. (**b**) MAP signature and STAD MSI signature on TCGA-STAD. (**c**) MAP signature and preMSIm signature on TCGA-COADREAD. (**d**) MAP signature and preMSIm signature on TCGA-STAD. (**e**) MAP signature and preMSIm signature on TCGA-UCEC. The x-axis represents log_2_ fold changes in gene expression data for MSI versus MSS samples. The colored dots mean the genes of the corresponding signatures marked in each panel. The blue dotted line on the x-axis means − 1 and 1 of the log_2_ fold change scale, and 2 (− log_10_(0.01)) on the y-axis.

Using TCGA-STAD and TCGA-UCEC RNA-seq datasets, we evaluated whether MAP, which was developed for CRC, could be applied to other cancers. In the stomach adenocarcinoma (STAD) and uterine corpus endometrial carcinoma (UCEC) data, accuracies of 80.2% and 75.4% were observed, respectively. To investigate why the MSI classifier of CRC is not suitable for other cancers, the same method used to construct the MSI signature (MAP signature) in CRC was applied to examine MSI signatures in gastric cancer and uterine cancer, and then the differences in expression patterns were investigated. Uterine cancer showed an accuracy of 90.9%, with only nine genes (*CXCL13, EPM2AIP1, H2AFJ, HOXA9, MLH1, RNLS, SDR42E1, TNFSF9*, and *ZNF300*), whereas gastric cancer reached an accuracy of 83.4% using 78 genes (Table S4). We further probed how cancer-specific MSI signatures are expressed in each cancer and observed that individual MSI signatures tend to correspond to DEGs not statistically significant in other cancers (Fig. 3a,b). *MLH1* and *EPM2AIP1* were differentially expressed in all three cancers, *RPL22L1* was included in the MAP signature of CRC and STAD, and *H2AFJ* was observed in both CRC and UCEC. In addition, comparing the MAP signature and MSI signature from the recently developed preMSIm, five genes (*MLH1, RPL22L1, EPM2AIP1, DDX27*, and *SHROOM4*) were observed in both signatures in CRC (Fig. 3c). It is also worth mentioning that all of the genes used in preMSIm are down-regulated in MSI, except *RPL22L1*, whereas the MAP signature additionally includes both up- and down-regulated genes in CRC. In addition, the expression pattern of the signature of preMSIm did not appear to suitably reflect genes important in gastric cancer, such as *DDX27, SMAP1*, and *ZSWIM3*, and in uterine cancer, such as *DDX27, SHROMM4, SMAP1*, and *ZSWIM3*, thereby making it unable to efficiently differentiate MSI and MSS tumors (|log_2_ fold change|< 0.5) in these cancer types (Fig. 3d,e).

## Discussion

Not all MSI status information is available in publicly available colorectal cancer expression data, such as NCBI Gene Expression Omnibus (NCBI GEO), thus such data cannot be utilized in MSI CRC research. For example, it hampers studies determining why most MSS samples belong to the immune desert type or the mechanism by which immune evasion occurred in a subset of MSI tumors by utilizing molecular or immunological characterization of MSI and MSS. Although at the research level, if these studies are conducted, this may give clues to convert the immune-inactivated tumors into immune-activated types or to discover drugs targeting abnormally activated oncogenic pathways or suppressed TMEs which can be combined with ICIs. Additionally, since MSI samples are rare, with the difficulty of producing expression data due to RNA degradation, meta-analysis using multiple cohorts is required, but the use is hindered due to the absence of MSI status information in them. Furthermore, MSI research analysis can be performed after cross-validation of the MSI status of RNA-seq data of the tumor identified as the MSI sample at the DNA level. Here, we present MAP, a tumor microenvironment-aware, single-transcriptome predictor of MSI in CRC, with robust accuracy validated using large samples from multiple cohorts of primary tumors. (*N* = 1118). We expect that the MAP will open the door to make such datasets of use in future MSI studies. MAP has the advantage of not requiring a matched normal sample as a control and sufficiently predicts MSI status with a single-sample transcriptome profile. In other words, to function as a single sample predictor, the ratio of gene pairs and the score of the signature composed of the tumor and TME characteristics were used as an input to the random forest models leveraging multiple decision trees. Therefore, MAP was an MSI predictor of RF model in which complex rules were comprehensively reflected rather than just a few rules.

Attempts to create an absolute predictor for subtype classification of cancer and stratification of patients by applying relationships or ratios between two genes, not the expression value of the gene itself, are ongoing^22,23^. MAP is an absolute classifier, not relative, and was developed to reflect tumor molecular characteristics, immune-related signatures, and tumor-infiltrating immune cells in TME of CRC. Also, since MAP is an RF classifier, one feature does not represent all MSI in common, but the MSI status is determined through the complex reflection of various features. Therefore, it may be difficult to interpret clinical and biological significance of features, and it might be considered to be included technical as well as biological rules to improve the accuracy of classification.

During the development, it showed an accuracy of 99.1% (1/115) in the correct identification of MSI in the internal validation TCGA dataset. Only one sample (TCGA-DC-6154) with MSI status was incorrectly predicted as being MSS by the MAP model, and it was also marked as MSS with the MOSAIC program, a tool which predicts MSI status at genomic level^10^. We speculated such discrepancy may stem from the different tissue sampling locations (MSI typing vs. DNA and RNA sequencing) or MSI intratumor heterogeneity, rather than MAP misinforming. We also encountered misclassification of a 11CO070 (MSS) hypermutated sample from an external RNA-seq validation dataset and five MSS samples from the GSE39582 dataset as MSI by MAP. Using the clinical information available, we further investigated the five MSS samples from the GSE39582 dataset and they all carried BRAF mutation and high CpG island methylator phenotype (CIMP). In sporadic MSI CRC, the accompanying characteristics of BRAF mutation and high CIMP are known to be strongly correlated with MSI^24^, but it was not possible to determine misassignment or tumor heterogeneity characteristics in detail due to the absence of lynch syndrome status or mutation information of other MMR genes of the samples. Additionally, in research on CMS reported by the Colorectal Cancer Subtyping Consortium, the distribution of CMS2 (known as immune-desert type) samples with MSI status was exceedingly rare (10 of 270, 2.7%)^2^, whereas eight out of the 10 CMS2-MSI samples belonged to one cohort (GSE13294 dataset). This particular cohort carried a slightly dissimilar CMS2-MSI population distribution from the other datasets, and out of these eight samples, five were classified as MSS by MAP.

MAP showed accuracies of 98.6% (95% CI 97.6–99.6) in RNA-seq and 95.1% (95% CI 91.6–98.7) in microarray data, all primary tumor and MSI detected based on PCR panel, showing a slight difference depending on the platform. Although MAP is an absolute SSP with a specificity of approximately 97% and a high accuracy of 96.1%, it may be due to the inherent characteristics derived from development based on RNA-seq, or a rare MSI subgroup (e.g., immune-desert CMS2-MSI) that exists in a specific cohort (GSE13294). Due to the paucity of clinical information, we were unable to thoroughly characterize the samples that were not accurately predicted.

The recently developed preMSIm, a pan-cancer MSI predictor, is a k-NN classifier using 15 genes identified by using only three frequent cancer types (COAD, STAD, and UCEC) as training data. However, due to the limitations mentioned by the author of preMSIm^12^ and based on our findings, these 15 genes are not enough to predict MSI in pan-cancer. This is because tumor biology and tumor microenvironment are distinct for individual cancer origins, suggesting diverse tumor-intrinsic gene expression patterns. In this context, MAP is superior when predicting the MSI status in CRC as it was designed to reflect both the molecular characteristics of CRC and the complexity of its surroundings.

*MLH1* was included in the list of MAP signatures used in the MAP model without other MMR genes, which implies that the TCGA data used as training data may include many tumors due to MLH1 deficiency. Thus, MAP enables classify sporadic CRC, characterized by *MLH1* promoter hypermethylation or *MLH1* loss, whereas Lynch syndrome, a familiar syndrome, due to germline mutations of MMR or *EPCAM* gene^1^, may not be reflected. In addition, due to the lack of IHC and clinical information (e.g., *KRAS, BRAF* mutations, and Lynch syndrome status) in the validation datasets, the characteristics of samples with incorrectly predicted MSI status (e.g., *MSH2/MSH6*-negative CRC) could not be thoroughly assessed. Although MAP reflects the characteristics of sporadic MSI CRC well, *MSH2/MSH6*-negative MSI CRC reflection is somewhat limited because the expression patterns of *MSH2* and *MSH6* among MMR genes are not distinctly distinguished from MSS and MSI in TCGA and external validation dataset and information on each MMR gene-negative phenotype was not available in the TCGA clinical information, so it was not considered during training. Finally, we built a model using TCGA CRC data as training data to use the most well-researched public data. However, because this study did not utilize training data that we could control or investigate in depth, the opportunity to link the model's results with biological features in detail was limited.

In conclusion, we provided MAP, an MSI predictor for CRC that is robust and accurate. Although MSI prediction based on IHC and PCR is well established and available at a low cost for clinical application, MAP will find use in MSI-related research seeking to employ the large amounts of publicly available CRC expression data and will be useful for gaining insights to help with translational medicine.

## Methods

### Dataset acquisition

This meta-analysis was performed in accordance with the PRISMA guidelines (Fig. S5). For the discovery cohort, 581 RNA-seq data (rsem.norm.expression) from TCGA-COADREAD were downloaded from the TCGA data portal (https://portal.gdc.cancer.gov/). Matching data on MSI status (82 MSI and 499 MSS) was downloaded from The Cancer Imaging Archive (TCIA) (https://tcia.at/). For the validation cohort, 106 RNA-seq data from 24 MSI and 82 MSS samples (rsem.norm.expression) from an independent study^25^ were downloaded. MSI-low tumors were grouped with MSS tumors as in previous studies^26,27^. The gene expression values of RNA-seq were log-transformed (with base 2) for analysis. Five independent microarray-based cohorts were used as an additional validation dataset, particularly to test platform compatibility^25,28–32^. Detailed information on the datasets is available in Table S1. Information on consensus molecular subtype (CMS) classification was obtained from the Colorectal Cancer Subtyping Consortium for all array datasets^2^. For cases with missing CMS information, CMS labels were inferred by using the random forest (RF) method provided by the CMSclassifier R package^2^. Genes covered in both of the discovery and validation datasets were used for further analysis.

### Development of the MAP predictor

#### Development of a gene-based predictor (MAPgene)

A schematic drawing of the MAP development process is provided in Fig. 1a. To select informative genes for MSI prediction, we first identified differentially expressed genes (DEGs) between MSI and MSS tumors using the Wilcoxon rank-sum test in the discovery cohort. To construct and train a prediction model, RNA-seq data were divided into training and internal validation datasets at a ratio of 4 to 1. To extract the most discriminative genes from the DEGs, the recursive feature elimination-random forest (RFE-RF) algorithm was used on the 466 training dataset. Briefly, feature selection was conducted by the backward selection method, wherein the RFE-RF repeatedly constructed an RF model by eliminating features with the least importance. The selection process was repeated 100 times, applying an upsampling approach to the MSI group (due to the small group size) using caret^33^ and randomForest R package. The final model (MAPgene) was then selected based on that with the best area under a receiver operating characteristic curve (AUC) for 31 genes.

#### Development of an absolute, gene-pair-based predictor (MAPpairs)

To make the MAPgene model absolute (i.e., to predict MSI status from a single patient without comparison to a reference cohort or sample-wise normalization), a new model (MAPpairs) was developed using pairwise gene expression values instead of single gene expression values. A total of 465 (_31_C_2_) gene-expression pairs were generated for the selected 31 genes. These gene expression pairs were then converted to rules that indicated the relative over- or under-expression between two genes. For example, if the expression of gene A was higher than that of gene B, the rule (gene A > gene B) was generated. Another RFE-RF model was then constructed using the 465 rules and trained with a five-fold cross-validation. Similar to the feature selection procedure, RFE-RF was applied with a five-fold cross-validation and repeated 100 times. The final absolute model was selected according to its AUC.

#### Development of a tumor microenvironment-integrated model (MAPsig)

To construct a more sophisticated model, we exploited the molecular differences in cancer-, immune-, and TME-related signatures between MSI and MSS tumors. We collected 101 signatures, including immune and stromal cells (TCIA and MCP-counter)^34,35^, cancer hallmarks from MSigDB^36^, immune-related signatures, such as epithelial and mesenchymal signatures^37^; stromal and immune signatures^38^; immunoinhibitory signatures and immunostimulatory signatures)^34^; T-cell-inflamed gene expression profile (GEP) ^39^ and IFN-γ expanded signatures ^39^; cell cycle signature^40^; cell cycle regulator^41^; mismatch repair (KEGG), C-ECM signature^42^; angiogenesis, HLA class I and II family signature^43^; pro-inflammatory cytokines and chemokines^43^; CD8 T cells (Teff)^44^; and the MAP signature. To obtain signature scores for each individual sample, single sample gene-set enrichment analysis (ssGSEA), with ssgsea.norm = F, was applied for the signatures above. Additionally, for cross-platform comparability, the acquired score was adjusted to a value between 1 and 10. We used the same modeling method as that for the MAPgene and MAPpairs models, although with different input values. Finally, the MAPsig model and features were selected for inclusion in the final according to those that provided the best AUC.

#### Model refinement

When applying the MAPpairs model, we noted that true MSI samples tended to be classified with MSI at a probability much higher than 70%. Thus, only samples with a probability of having MSI that was more than 70% were assigned MSI status. Samples with a predicted probability of MSI that was lower than 70% were further examined by applying the MAPsig model to determine final MSI status, as it showed high overall AUC, accuracy, and specificity, but low sensitivity, making it of use in only verifying a true MSS sample. The software is available at https://sourceforge.net/p/mapmsi/wiki/MAP/.

### Validation dataset

To evaluate the predictive performance of the MAP model, we employed RNA-seq data for CRCs (*N* = 106) with log_2_-transformed rsem.norm data. Also, to assess platform independency and the applicability of MAP on different array datasets, we collected data for five cohorts. In the microarray datasets, the probes per gene were selected using Jetset (http://www.cbs.dtu.dk/biotools/jetset/)^45^. The array datasets were processed using fRMA R package per sample^46^. A total of five datasets were evaluated for the following: accuracy, sensitivity, specificity, F1 score, and balanced accuracy. All information on the datasets is provided in Table S1. For RNA-seq of stomach adenocarcinoma (STAD), and uterine corpus endometrial carcinoma (UCEC) were downloaded from the TCGA data portal (https://portal.gdc.cancer.gov/).

### Consistency of genes in a microsatellite instability classifier model based on gene expression

To verify the consistency of feature genes with discriminative value in an MSI classifier model using gene expression, the Wilcoxon rank-sum test was used to analyze the external RNA-seq validation dataset. In addition, to assess the utility of MAP for MSI prediction, we calculated MAP signature scores (31-gene-set signature) using ssGSEA and compared them between MSI and MSS groups, as well as among MSI CMSs, using the Wilcoxon rank-sum test and Kruskal–Wallis test.

### MSI signature construction at UCEC and STAD

To investigate the MSI signature that can distinguish MSS and MSI in each cancer types, the same method was applied when constructing the MAP signature, except that the *P* < 0.02 and |log_2_ fold change|> 1 criteria was applied to identify sufficient number of DEG from two types of cancer. TCGA-UCEC and STAD expression dataset were download TCGA-UCEC and STAD RNA-seq data were downloaded from EBPlusPlusAdjustPANCAN_IlluminaHiSeq_RNASeqV2.geneExp.txt file at https://gdc.cancer.gov/about-data/publications/panimmune. In this file, only 12 of 15 signatures of preMSIm existed. The missing genes were *HENMT1, NOL4L*, and *RTF2*.

### Correlation analysis of MSI status with TME and oncogenic signatures

To investigate the effect of MSI status on TME and oncogenic signatures, Spearman correlation analysis was performed between the MSI probability of each sample from the MAPsig model and the signature features of the MAPsig model. Statistically significant features have *P* < 0.01 and |correlation coefficient (rho)|> 0.3.

### Statistical analysis

Comparisons of two groups were conducted using the Wilcoxon rank-sum test, while comparisons of multiple groups were performed using the Kruskal–Wallis test. All statistical analyses were conducted using R language software (https://www.r-project.org/).

### Ethics approval and consent to participate

Not applicable. No permissions were required to use any of the repository data. All methods were performed in accordance with the PRISMA guidelines.

## Supplementary Information

Supplementary Information.

## Data Availability

All relevant datasets used in the current study are available in the TCGA (https://www.cancer.gov/about-nci/organization/ccg/research/structural-genomics/tcga) and GEO (https://www.ncbi.nlm.nih.gov/geo/). This study analysis used all publicly available datasets, and the dataset accession numbers included in Table S1. The software is available at https://sourceforge.net/p/mapmsi/wiki/MAP/.
