# Supplementary material for: Tumor microenvironment-aware, single-transcriptome prediction of microsatellite instability in colorectal cancer using meta-analysis
Source: Sci Rep. 2022 Apr 15;12:6283. doi: 10.1038/s41598-022-10182-3 (PMC9012745; doi:10.1038/s41598-022-10182-3)
Supplement: Supplementary file 1 — Supplementary Information. [file 41598_2022_10182_MOESM1_ESM.pdf]

Supplementary Information

Tumor microenvironment-aware, single-transcriptome prediction of microsatellite instability in colorectal cancer using meta-analysis

Mi-Kyoung Seo[1], Hyundeok Kang[1], Sangwoo Kim[1*]

Supplementary material

Supplementary Figures

Figure S1 Boxplot of MMR gene expression.
Figure S2 Distribution of features in the MAPpairs model.
Figure S3 Heatmap of 44 features in the MAPsig model.
Figure S4 Barplot for correlation of features and probability for MSI in the MAPsig model.
Figure S5 PRISMA Flow Diagram.

Supplementary Tables

Table S1 The dataset used in this study
Table S2 Results of the MAP model in the validation datasets
Table S3 The MAP signatures
Table S4 The MSI signature in STAD, UCEC and preMSIm

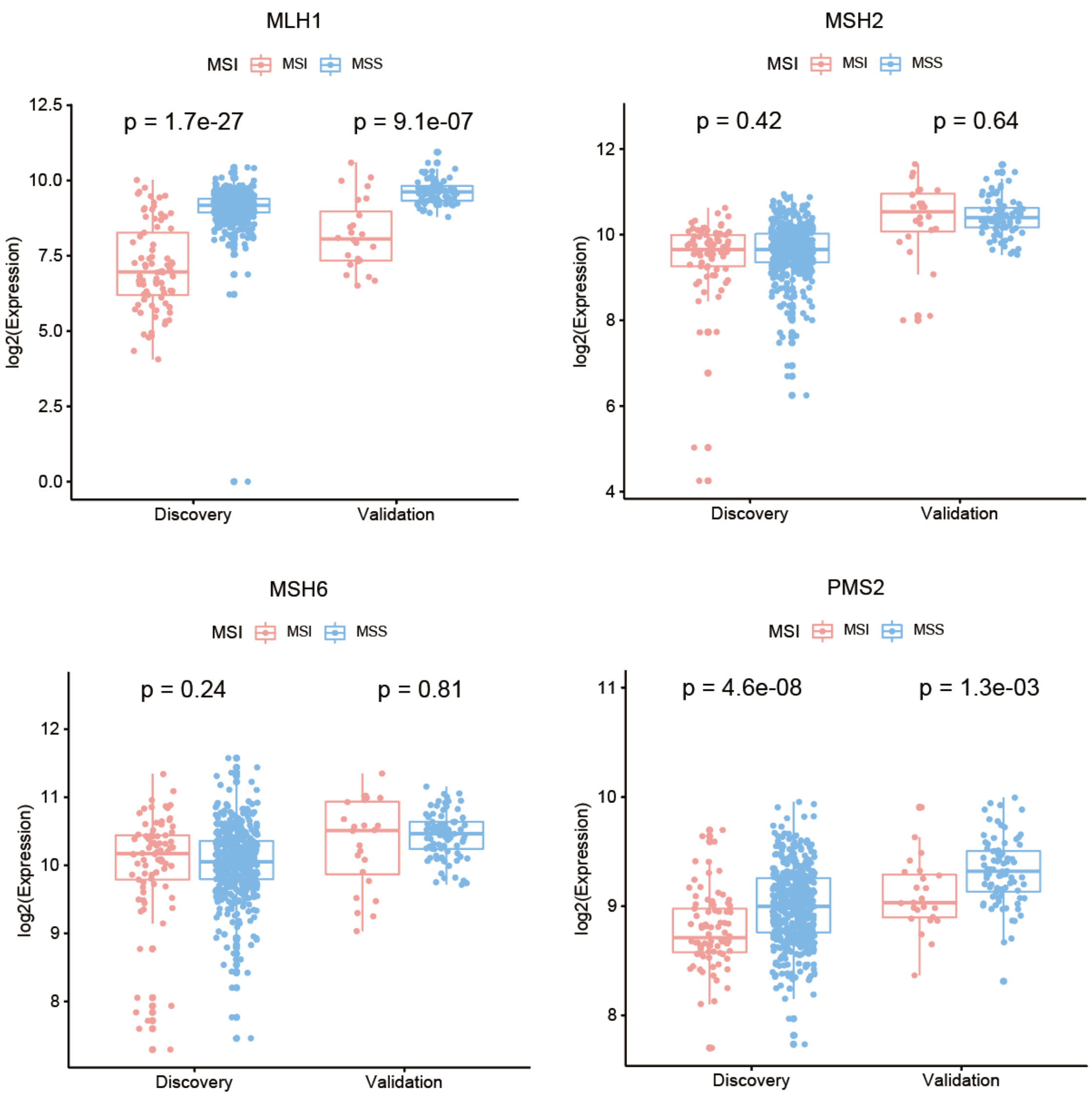

Figure S1. Boxplots of MMR gene expression. Boxplots of the gene expression of four genes implicated in mismatch repair (MMR) for the Discovery (TCGA-CRC) and RNA-seq validation datasets (Vasaikar *et al.*). Blue dots indicate MSS tumors and pink dots indicate MSI tumors.

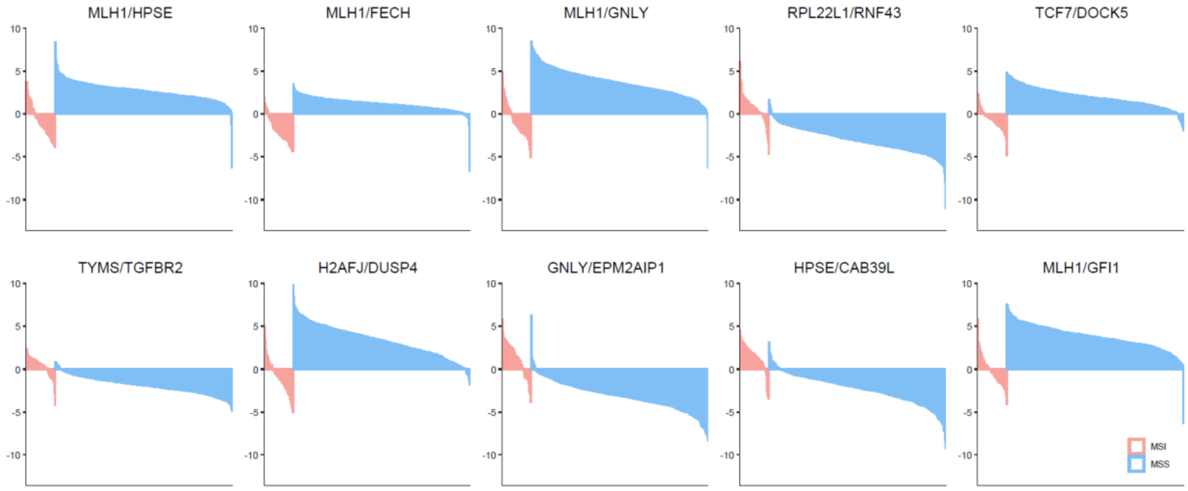

Figure S2. Distribution of features in the MAPpairs model. The y-axis means the ratio of two genes (feature) in each sample. Blue indicates MSS and pink indicates MSI tumors.

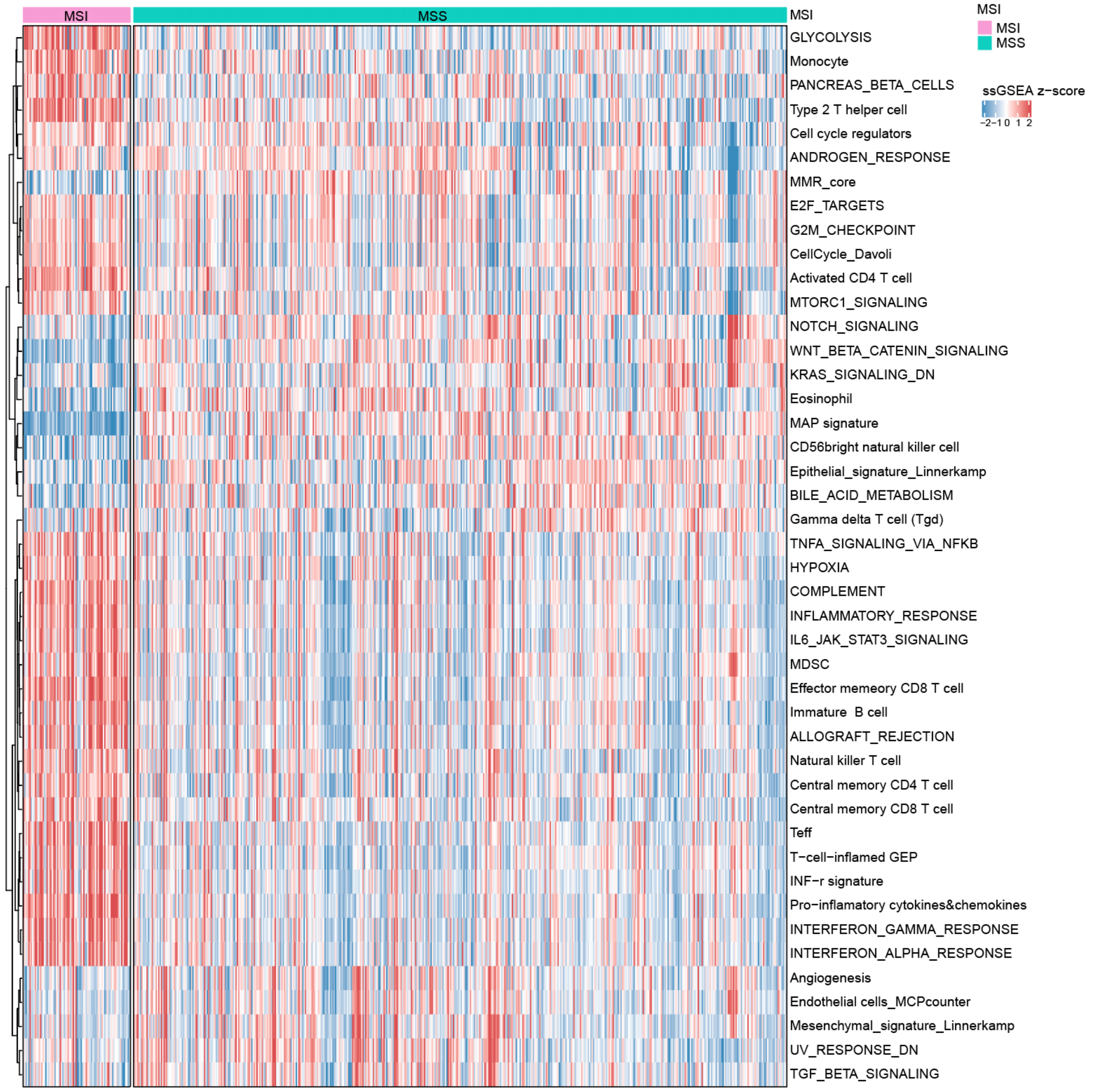

Figure S3. Heatmap of 44 features in the MAPsig model. Heatmap was based on the inferred ssGSEA score for the 44 features of the MAPsig model. *ssGSEA ; single-sample gene set enrichment analysis. The figure was created using the ComplexHeatmap (v2.2.0) package with R (v3.6.0) software.

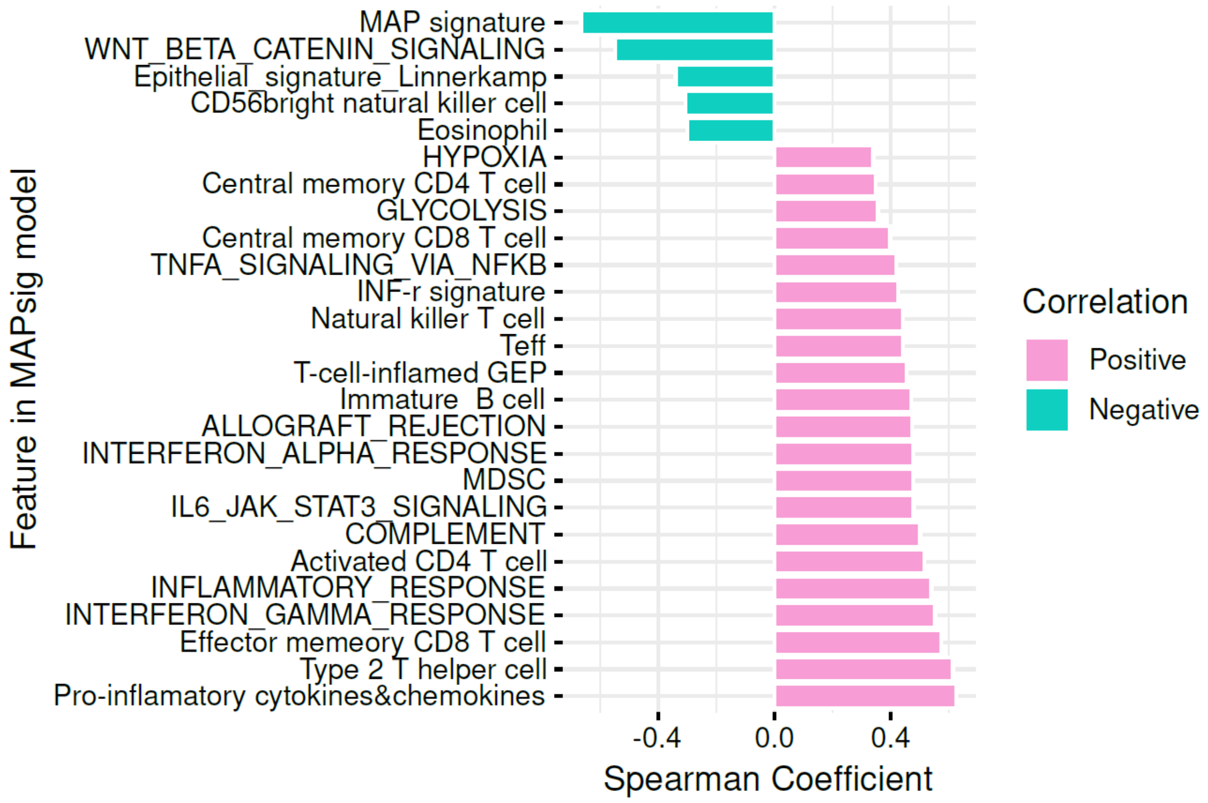

Figure S4. Barplot for correlation of features and probability for MSI in the MAPsig model. The figure was created using the ggpubr (v0.4.0) package with R (v3.6.0) software.

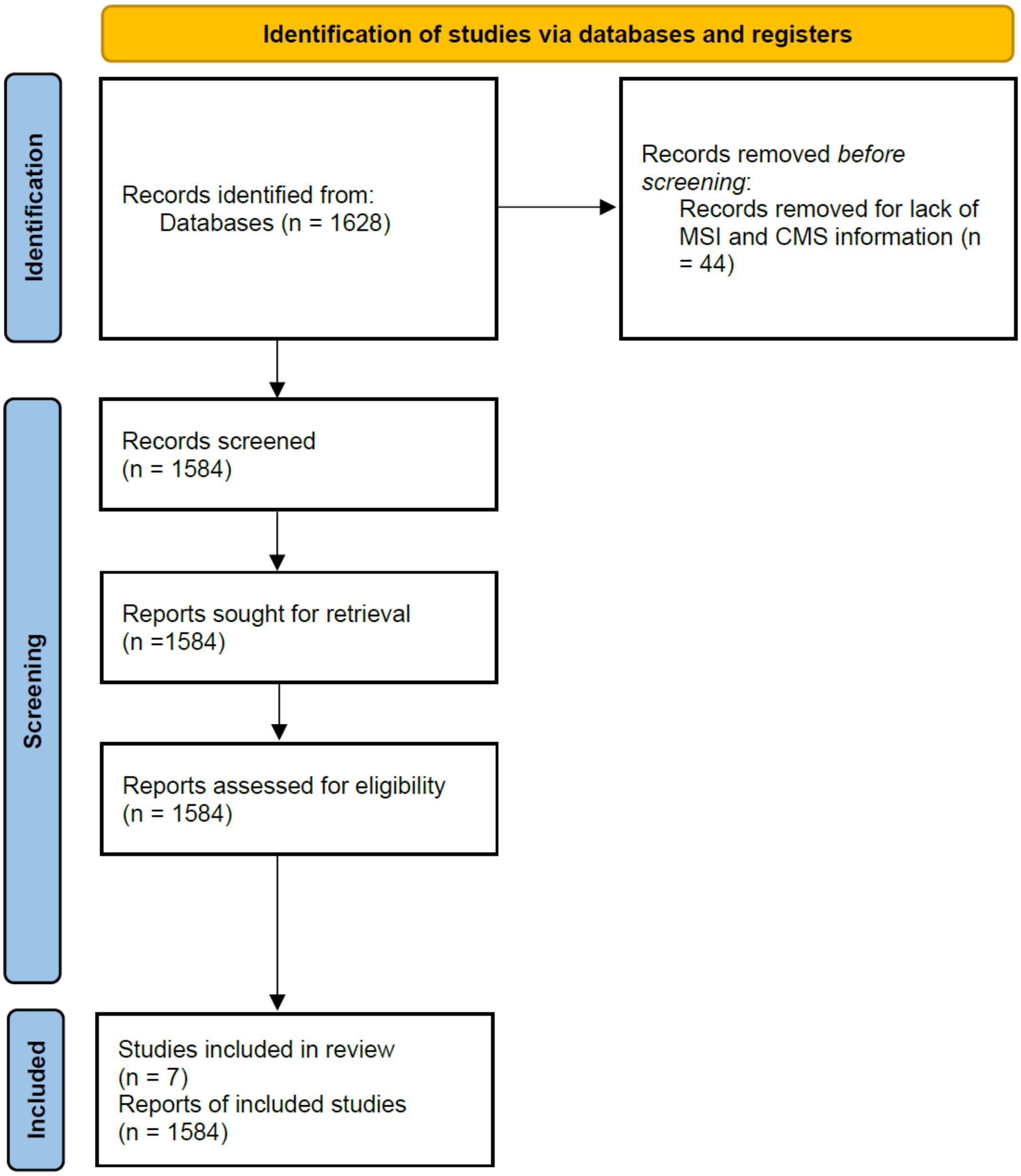

Figure S5. PRISMA Flow Diagram.

Table S1. The dataset used in this study

Purpose	Dataset	Platform	Assay to detect MSI status	No. of MSI	No. of MSS	No. of total samples	CSCSC
Training	TCGA-CRC	RNA-seq	MSI-Mono-Dinucleotide assay	66	400	466	Y
Internal validation	TCGA-CRC	RNA-seq	MSI-Mono-Dinucleotide assay	16	99	115	Y
External validation	Vasaikar *et al.* (2019)	RNA-seq	MSI Analysis System Version 1.2 (Promega)	24	82	106	N
External validation	GSE13067	Array, HG-U133_Plus_2	Bethesda microsatellite panel	11	63	74	Y
External validation	GSE13294	Array, HG-U133_Plus_2	Bethesda microsatellite panel	78	77	155	Y
External validation	GSE33113	Array, HG-U133_Plus_2	MSI Analysis System Version 1.2 (Promega)	25	65	90	Y
External validation	GSE39582	Array, HG-U133_Plus_2	Bethesda microsatellite panel	75	444	519	Y
External validation	GSE75316	Array, HG-U133_Plus_2	NA	11	48	59	N
Total validation set			-	240	878	1118	-
Total			-	306	1278	1584	-

In the GSE75316 data, MSI status information was present, but information on the assay that detected it was not provided, so it was marked as NA (not available). The CRCSC column of the table indicates whether CRCSC was included in constructing the CMS classification system.
* CRCSC ; Colorectal Cancer Subtyping Consortium, CMS ; consensus molecular subtype.

Table S2. Results of the MAP model in the validation datasets

Purpose	Platform	Dataset	Accuracy	Kappa	Sensitivity	Specificity	F1	Balanced Accuracy
Internal validation	RNA-seq	TCGA	99.13 (95.25-99.98)	96.27	93.75	100	96.77	96.88
External validation	RNA-seq	Vasaikar *et al.* (2019)	98.11 (93.35-99.77)	94.77	100	97.56	96	98.78
	Array	GSE13067	98.65 (92.70-99.97)	94.85	100	98.41	95.65	99.21
	Array	GSE13294	90.32 (84.54-94.48)	80.67	82.05	98.7	89.51	90.38
	Array	GSE33113	93.33 (86.05-97.51)	84.16	96	92.31	88.89	94.15
	Array	GSE39582	93.26 (90.75-95.26)	73.46	80	95.5	77.42	87.75
	Array	GSE75316	100 (93.94-100)	100	100	100	100	100
average			96.11 (94.34-98.87)	89.17	93.11	97.5	92.03	95.31
average/rnaseq			98.62 (97.62-99.61)	95.52	96.88	98.78	96.39	97.83
average/array			95.11 (91.55-98.65)	86.63	91.61	96.98	90.29	94.3

In the table, the numbers in parentheses in the accuracy column represent 95% confidence intervals.

Table S3. The MAP signatures

Symbol	P-value	FDR	MSI median	MSS median	log_2 Fold-change
LY6G6D	2.02E-36	2.29E-32	0.72	8.48	-7.77
CYP2W1	8.03E-20	2.77E-18	4.19	8.65	-4.46
TNNC2	4.78E-32	2.71E-29	2.72	7.11	-4.4
CTTNBP2	8.79E-32	4.33E-29	4.32	7.82	-3.5
NKD1	2.67E-28	6.18E-26	5.97	9.22	-3.25
CAB39L	3.38E-33	4.79E-30	6.37	8.58	-2.21
MLH1	3.58E-28	7.50E-26	6.97	9.18	-2.21
EPM2AIP1	5.50E-27	8.54E-25	6.65	8.68	-2.03
SHROOM4	5.61E-33	6.13E-30	7.12	9.13	-2.02
RNF43	9.45E-34	1.78E-30	10.72	12.62	-1.91
PRR15	6.98E-34	1.77E-30	9.48	11.09	-1.6
ATP9A	8.97E-33	8.47E-30	10.66	12.18	-1.53
H2AFJ	4.95E-21	2.19E-19	9.4	10.92	-1.52
FARP1	4.49E-32	2.68E-29	9.63	11.04	-1.41
TCF7	1.14E-32	9.92E-30	8.87	10.26	-1.38
MAPRE3	1.99E-25	2.25E-23	7.12	8.4	-1.28
ZMYND8	3.54E-32	2.35E-29	9.53	10.78	-1.25
DDX27	3.15E-32	2.23E-29	9.9	11.12	-1.22
TGFBR2	2.21E-22	1.27E-20	10.32	11.51	-1.19
PIWIL4	3.50E-25	3.64E-23	6.45	7.63	-1.18
FECH	8.36E-32	4.31E-29	9.09	7.94	1.15
DOCK5	7.96E-28	1.61E-25	9.49	8.32	1.17
TYMS	3.73E-32	2.35E-29	10.92	9.58	1.35
HPSE	2.83E-30	1.00E-27	8.03	6.49	1.54
ASPHD2	2.24E-33	3.63E-30	8.82	7.18	1.64
AGR2	2.03E-23	1.42E-21	15	13.16	1.84
GFI1	2.91E-28	6.47E-26	7.37	5.34	2.03
RPL22L1	3.44E-28	7.35E-26	11.47	9.39	2.08
RAB27B	4.87E-33	6.13E-30	6.79	4.08	2.71
GNLY	2.87E-28	6.47E-26	8.31	5.49	2.82
DUSP4	7.27E-29	2.06E-26	10.33	7.49	2.85

Comparisons of MSI vs. MSS groups were conducted using the Wilcoxon rank sum test. Fold-change means log2 (MSI/MSS). Blue genes are down-regulated and red are genes up-regulated in MSI compared with MSS.

Table S4. The MSI signatures in STAD, UCEC and preMSIm

MAP_STAD		MAP_UCEC	preMSIm_15 gene-set
MLH1	PPP1R1B	MLH1	DDX27
EPM2AIP1	DTX3	EPM2AIP1	EPM2AIP1
F12	CXCL14	RNLS	HENMT1
RPL22L1	ZNF134	H2AFJ	LYG1
GSTA4	CES3	CXCL13	MLH1
HOXA11	DNASE1L3	ZNF300	MSH4
PLEKHH2	SHROOM2	HOXA9	NHLRC1
GAMT	ZNF461	TNFSF9	NOL4L
SCAMP5	TMEM52	SDR42E1	RNLS
IRAK3	WNT4		RPL22L1
ZNF606	SERP2		RTF2
EFNA3	POLR3G		SHROOM4
TNFSF9	RBP7		SMAP1
KRT23	GPR143		TTC30A
PLEKHB1	C3orf14		ZSWIM3
ALDH1A1	GPC3		
NMU	METTL7A		
HOXA10	CARD11		
GPRC5B	VANGL2		
FUZ	EPHA4		
GPA33	COL11A1		
F10	KIAA1257		
TCEA2	PON3		
RGS5	HOXA9		
ZNF790	APOD		
CTSF	CASP5		
RBP1	FRZB		
GSTA1	NUP210		
HOXA13	FAM84A		
CPNE7	FBXO17		
ZNF43	NOS2		
IL34	ZSCAN18		
PBK	SYT13		
NHLRC1	CCDC106		
RAB37	QPRT		
ENPP5	ANKRD29		
TMPRSS13	PFN2		

SPAG16	DNAJC12
TPPP3	ZNF300
